# Jejunojejunal intussusception secondary to inflammatory fibroid polyp: A rare cause of small bowel obstruction

**DOI:** 10.1016/j.amsu.2020.10.009

**Published:** 2020-10-10

**Authors:** Jun Sam Tan, Kai Ming Teah, Vee Chuan Hoe, Allim Khairuddin, Harivinthan Sellapan, Firdaus Hayati, Nor Haizura Ab Rani

**Affiliations:** a Department of Surgery, Queen Elizabeth Hospital, Ministry of Health Malaysia, Kota Kinabalu, Sabah, Malaysia; b Department of Anaesthesiology, Queen Elizabeth Hospital, Ministry of Health Malaysia, Kota Kinabalu, Sabah, Malaysia; c Department of Surgery, Faculty of Medicine and Health Sciences, Kota Kinabalu, Sabah, Malaysia; d Department of Pathology, Queen Elizabeth Hospital, Ministry of Health Malaysia, Kota Kinabalu, Sabah, Malaysia

**Keywords:** Benign neoplasms, Inflammatory polyps, Intussusception, Jejunum

## Abstract

**Background:**

Adult intussusception is a relatively rare clinical entity. The majority of cases of intussusception in adults are due to a pathologic condition that serves as a lead point and requires surgery. Small bowel intussusception is usually caused by benign or malignant neoplasms appearing at the head of the invagination. Inflammatory fibroid polyp (IFP) of the small bowel is an unusual benign neoplastic lesion that has been rarely reported to cause intussusception, especially in the jejunum.

**Case presentation:**

We present a rare case of adult intussusception who presented with a triad of intestinal obstruction. Computed tomography revealed small bowel intussusception with bowel ischemia. Intraoperatively, she required resection of the small bowel and primary anastomosis. Macroscopic examination revealed a single pedunculated polyp, which is the lead point of intestinal obstruction and confirmed histologically.

**Conclusion:**

Inflammatory fibroid polyp should be considered as a cause of intussusception among adults with small bowel obstruction.

## Highlights

• IFP must be considered among adults with small bowel intussusception.
• Surgery is the mainstay of treatment in obstructed small bowel intussusception.
• Histological examination with immunohistochemistry is warranted to establish the final diagnosis.

## 1 Introduction

Intussusception is defined as the telescoping of a segment of the gastrointestinal tract (the intussusceptum) in the lumen of the adjacent segment (the intussuscipiens) [1]. Adult intussusception is very rare, demonstrating 5% of all intussusception cases and accounts for 1%–5% of intestinal obstruction in adults [1]. Inflammatory fibroid polyp (IFP) is one probable rare cause of an intussusception and bowel obstruction. They are usually benign, tumorous lesions of the gastrointestinal tract. The stomach is the most common site of IFP, followed by the small bowel, and, more rarely, the large bowel, duodenum, gallbladder, and oesophagus [2]. In this case report, we report a rare case of jejunojejunal intussusception caused by an IFP. This work has been reported in line with the SCARE criteria [3].

## 2 Presentation of case

**Fig. 1:**
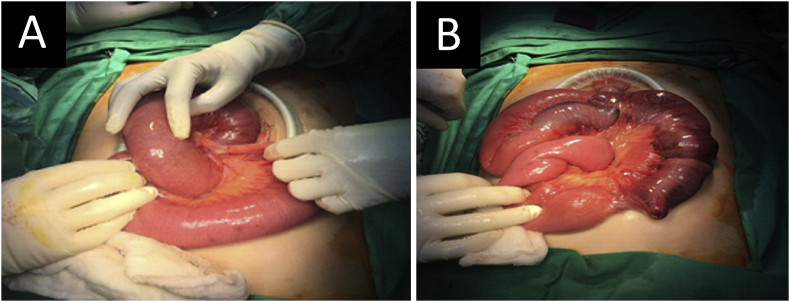
(A) Intraoperative finding showing a jejunojejunal intussusception with dilated proximal small bowel. (B) Gangrenous bowel found after reduction.

A 20-year-old lady with no known medical illness, drug intake or genetic history presented with a triad of intestinal obstruction for two days. Physical examination revealed generalised abdominal tenderness with guarding. The abdominal radiograph showed a dilated small bowel. Computed tomography (CT) of the abdomen revealed a small bowel intussusception with evidence of bowel ischemia. During the emergency laparotomy by the attending surgeon, jejunojejunal intussusception was found at 25 cm from the duodenojejunal junction with a 40 cm length of the gangrenous small bowel (intussuscipiens) (Fig. 1a). The macroscopic examination of the surgical specimen revealed a pedunculated polyp measuring 2 × 2 cm in diameter with a long stalk. In view of a gangrenous bowel, bowel resection was performed with primary anastomosis (Fig. 1b). He recovered well postoperatively.

**Fig. 2:**
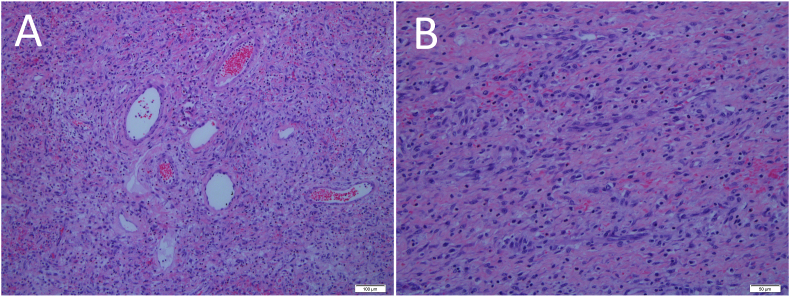
(A) Histopathological examination showing tumour composed of small vessels with perivascular spindle cells (H&E stain, original magnification ×10). (B) Microscopic findings of variable cellularity, and spindle cells with bland nuclei, and clear cytoplasm with eosinophil infiltrate (H&E stain, original magnification ×20).

The histological section of the resected small bowel showed an extensive area of oedematous and congested submucosal with the presence of thromboemboli. An area with mucosal haemorrhage and necrosis was identified as well. The polypoidal lesion section showed inflammatory infiltrates with perivascular spindle cells, and eosinophil predominance without malignancy was seen (Fig. 2). Upon applying the immunohistochemistry (IHC), the spindle cells were immunoreactive for CD34 and negative for CD117 suggestive of IFP. The patient had recovered well postoperatively as she was counselled for early mobilization and chest physiotherapy. Upon clinic follow up at one month, he recovered well with no postoperative complication or recurrence. She expressed her gratitude to the attending team members including the surgeon, anaesthetist, medical officers and supporting staffs for achieving her great recovery.

## 3 Discussion

IFP is an unusual benign lesion of the gastrointestinal tract. It can develop in different gastrointestinal tract sites but is very rare in the jejunum (2). Any age group can be affected, but the peak incidence is between the sixth and seventh decades. Mostly, the lesions of IFP usually measure from 3 to 4 cm in diameter (2). Our patient has a smaller lesion of 2 × 2 cm in size but has caused a significant complication to the patient. The pathophysiology of IFP is unknown, but recent studies have revealed that IFP harbours a mutation of the PDGFRA gene being in favour of a neoplastic origin [4].

It is often asymptomatic, but can manifest as abdominal pain, vomiting, change of bowel habits, weight loss, and gastrointestinal bleeding. These symptoms are not as classical as in children. Decision-making among adults is limited and involves complementary imaging support especially in obstruction, perforation, and abscess. The use of abdominal radiography and ultrasonography is necessary, but CT is currently the confirmatory tool with sensitivity between 86 and 100% [5]. Our patient presented with classical presentations of intestinal obstruction and was diagnosed via CT scan.

Differential histopathological diagnoses include inflammatory myofibroblastic tumour (IMT), schwannoma, spindle-cell carcinoids, and gastrointestinal stromal tumour (GIST). The diagnoses are challenging as they regularly present as polypoid masses and intussusception similar to IFP. Microscopically, IFP is composed of mononuclear, spindle-shaped cells, forming a whirl-like structure. The inflammatory infiltration also includes blood vessels, lymphocytes, eosinophils, macrophages, and mastocytes. Instead, IMT is characterised by myofibroblastic spindle cells with inflammatory infiltrates of plasma cells and intermingled mast cells [6].

The acceptable treatment of intussusception remains unclear whether to subject for primary en-bloc resection or initial reduction followed by a limited resection [5]. In general, any patient with intestinal obstruction warrants a surgical resection. However, the decision for oncologic or non-oncologic bowel resection is debatable. High-risk patients, elderly, and malignant presentations must follow the cancer pathway and vice versa. A young patient with a low risk for malignancy, a non-oncologic resection was performed in our case.

## 4 Conclusion

IFP can manifest as a small bowel intussusception, especially when it involves young patients with low malignancy risk involving the small bowel. CT abdomen is a useful preoperative tool to diagnose and histological examination with IHC will help to establish the final diagnosis.

## Ethical approval

Not related as this is a case report.

## Source of funding

There are no sources of funding or financial support.

## Authors’ contributions

JST - literature search, manuscript draft and conceptual design of the work.

KMT - involvement in the management of the patient.

VCH - literature search.

AK - manuscript draft and involvement in the management of the patient.

FH - contributed the conceptual design of the work.

HS - involvement in the management of the patient.

NHAR - preparation of the histopathological figure and input.

## Guarantor

Firdaus Hayati.

## Consent

Written informed consent was obtained from the patient for publication of this case report and accompanying images. A copy of the written consent is available for review by the Editor-in-Chief of this journal on request.

## Provenance and peer review

Not commissioned, externally peer-reviewed.

## Abbreviation

IFP: Inflammatory fibroid polyp
CT: Computed tomography
IMT: Inflammatory myofibroblastic tumour
GIST: Gastrointestinal stromal tumours
